# Stability control for breath analysis using GC-MS

**DOI:** 10.1016/j.jchromb.2018.08.024

**Published:** 2018-10-15

**Authors:** X. Rosalind Wang, Julie Cassells, Amalia Z. Berna

**Affiliations:** a CSIRO Data61, PO Box 76, Epping, NSW 1710, Australia; b CSIRO Health and Biosecurity, GPO Box 1700, Canberra, ACT 2601, Australia; c Department of Pediatrics, Washington University School of Medicine, St. Louis, MO 63110, USA

**Keywords:** Stability, Variability, Day of analysis, Gas chromatography, Mass spectrometry, Breath, Thioethers

## Abstract

Gas chromatography mass spectrometry (GC-MS) instruments provide researchers and clinicians with a vast amount of information on sample composition, thus these instruments are seen as gold standard in breath analysis research. However, there are many factors that can confound the data measured by GC-MS instruments. These factors will make interpretation of GC-MS data unreliable for breath analysis research. We present in this paper detailed studies of two of these factors: instrument variation over time and chemical degradation of known biomarkers during storage in sorbent tubes. We found that a single quadrupole MS showed larger variability in measurements than a quadrupole time-of-flight MS when the same mixture of chemical standards was analysed for a period of up to 8 weeks. We recommend procedures of normalising the data. Moreover, the stability studies of breath biomarkers like thioethers, previously found indicative of malaria, showed that there is a need to store the samples in sorbent tubes at low temperature, 6 °C, for no more than 20 days to avoid the total decay of the chemicals.

## 1 Introduction

The study and analysis of exhaled breath is an attractive and promising area of metabolomics and personalised medicine. Firstly, breath is totally non-invasive and safe, making it much easier to collect than bio-fluids such as blood and urine. Secondly, breath carries a large number of volatile metabolites [1,2] and non-volatile compounds [[3], [4], [5], [6]], potentially providing researchers with relevant biochemical information. However, variations in sample collection, storage and analysis between different studies means it is difficult to compare the results. Therefore, standardising of methodologies will be necessary [7].

Gas chromatography is seen as a gold standard in analysis of volatile compounds in breath research [8]. When coupled with mass spectrometry (GC-MS), these instruments provide researchers and clinicians with a vast amount of information about sample composition, which gives clues to the biochemical processes in the body. Breath samples can contain over a thousand compounds, and the fragmentation of the compounds by GC-MS instruments generates mass spectrometric fingerprints that can be searched using spectral libraries, such as that from the National Institute of Standards and Technology (NIST), to identify the detected compounds.

Much has been discussed about the difficulties of using GC-MS instruments for breath analysis in areas of standarisation, different instruments, collection materials etc. [7]. However, there have been very few works concerning the problematic fact of instrument variability that may lead to the erroneous interpretation of results.

Instrument variability, over a period of time, is an important issue to consider in any study. These ‘*day of analysis*’ phenomena occur in many different instruments, where the same instrument outputs different values for the same concentration of compounds analysed over different days. Changes of instrument sensitivity can impact on chromatographic data and the interpretation of results. Thus, determining GC-MS accuracy and precision over time and how to use this information to correct for possible changes is vital to the results of any experiment and clinical trials. To our knowledge, this issue has not been addressed systematically for GC-MS instruments, with most reported results from these instruments using just the raw measurements for comparison [9]. In addition to intra-instrument variability, there is also a need to understand inter-instrument variability for the comparison of data gathered from different instruments, whether the instruments are of the same type of mass analyser or not. Furthermore, variabilities of sample collection setup, environmental conditions (temperature, humidity etc), patient-to-patient variability also affects the concentration of VOCs in the exhaled breath collected [10].

Appropriate methods to collect exhaled breath and adequate storage time and temperature are critical for the success of breath analysis [7]. One of the most common methods to concentrate and store breath samples are sorbent tubes. Sorbent tubes contain various types of solid adsorbent materials to best absorb volatiles of interest, they have been widely used in a number of breath studies [11]. While there have been several studies on the effect of storage in sorbent tubes on VOCs [[12], [13], [14], [15]], very few studies have taken into account the storage time and temperature needed to preserve the captured exhaled breath volatile (EBV) [15,16].

In our previous work, we discovered that the concentration of four sulphur-compounds increased in the breath of volunteers that underwent a controlled human malaria infection in a clinical trial of a new drug treatment [17]. These sulphur-compounds (thioethers) were detected at the earliest stages of infection when blood smear microscopy is unable to detect the malaria infection and when participants were asymptomatic. Thioethers are relatively unstable compounds compared to many other breath volatiles. They have low boiling points (88–90 °C) and chemically reactive double bonds. Moreover, the breath samples often need to be transported over long distances and stored for a variable number of days prior to analysis. Given the potential importance of the levels of thioethers in breath samples, there is a need to understand their stability in sorbent tubes from the time of collection to analysis. So far contradictory studies have shown that three out of the four thioethers were stable at 4 °C for up to 31 days [16] while others have shown that the stability of sulphur compounds in sorbent tubes is very poor [18]. We therefore need to understand the stability of the thioethers on sorbent tubes between the time of collection and analysis.

We present the results from the instrument stability studies of the two types of mass analysers: a single-quadrupole GC-MS instrument, and Quadrupole-Time-of-Flight MS instrument. By analysing tubes containing the exact same amount of the chemical mixtures over a period of days, we aim to determine the variability of the instruments' measurements and address systematically how to compare samples measured on different days and different instruments. We also present results of the stability of thioethers on sorbent tubes, appropriate storage and temperature and understanding the decay characteristics of the chemical are also critical for the success of breath analysis [7].

## 2 Material and methods

### 2.1 Mass analyser

Two types of mass analysers were used in our study: a single-quadrupole Gas Chromatography Mass Spectrometry (GC-MS) instrument, and a Gas Chromatography Quadrupole-Time-of-Flight Mass Spectrometry (GC-QTOF-MS) instrument. Compound samples, spiked onto sorbent tubes Tenax TA 35/60 and Sulficarb 40/70 (Markes International Limited, UK), were analysed on both instruments using thermal desorbers attached to the instruments.

The *GC-MS* instrument is a Bruker Scion MS Model 451 GC, coupled with a 1200 mass selective detector. Tubes were thermally desorbed in a TD100 thermal desorber (Markes International, UK). The tubes were first dry purged for 1 min at a flow of 20 mL/min then pre-purged for 1 min at a flow of 3.3 mL/min. The tubes were then thermally desorbed for 15 min at 280 °C and transferred to a cold trap (Inert Sulphur trap, Marks International, UK) held at 30 °C. The cold trap was subsequently heated to 300 °C and held for 5 min. The trap flow of 37 mL/min, split flow of 3.3 mL/min and column flow of 0.8 mL/min, resulted in a split ratio of 5.1:1 after the cold trap.

The GC was equipped with a ZB-5MS (Bruker Corporation, USA) fused silica capillary column (30 m × 0.25 mm, 0.25 μm film thickness) with He as the carrier gas (0.8 mL/min). The oven temperature of the GC column was programmed to rise from 35 °C (held for 5 min) and ramped to 250 °C at 5 °C/min. The final temperature of 250 °C was held for 2 min. The total run time for the analysis was 50 min and a solvent delay time of 2 min was used at the start of the run. The solvent delay time was used because all standards were diluted in methanol, the delay was used to avoid the large methanol peak that comes out early in the run. Mass spectrometry was performed in full scan electron impact mode at 70 eV scanning over the range m/z = 35–350 Da (scan time = 250 ms) with positive polarity.

The *GC-QTOF-MS* instrument consisted of a 789B Series GC and a 7200 QTOF MS (Agilent Technologies, USA) with EI. For the GC-QTOF-MS analysis, the tubes were thermally desorbed using a Unity2 with an Ultra2 autosampler (Markes International, UK). The tubes were prepurged for 1 min at a flow of 3.5 mL/min. The subsequent thermal desorption and cold trap transfer are the same as in the GC-MS setup. The cold trap flow of 37 mL/min, split flow of 3.5 mL/min and column flow of 1.9 mL/min, resulted in a split ratio of 2.8:1 after the cold trap.

The GC was fitted with a HP-5MS UI capillary column (Agilent J&W GC Column, 30 m × 0.25 mm, 0.25 μm film thickness) with He as the carrier gas supplied under constant pressure (20 psi, with a starting column flow of 1.9 mL/min). The oven temperature, total run time and solvent delay time was the same as in the GC-MS setup.

The quadrupole was set to a temperature of 150 °C and the collision cell had a nitrogen flow of 1.5 mL/min. The source was set to 230 °C, the emission current was fixed at 35 μA and the electron energy at 70 eV. The mass range was scanned from 35 to 350 Da at an acquisition rate of 5 spectra/s and scan time of 200 ms/spectra.

### 2.2 Stability study experimental setup

To measure GC-MS and GC-QTOF-MS instrument stability, we used a set of ‘standard’ chemicals (EPA 8240B Calibration Mix) over a number of weeks. Table 1 shows the chemicals used and their main chemical properties, and Table S1 shows the chemicals' approximate retention times. The mixture comes in as 1 mL vial at 2000 μg/mL, and was purchased from Sigma-Aldrich (Australia). The mass and ions for the chemicals are from the NIST library.

**Table 1:** The compounds used for instrument stability.

Compound	Symbol	Exact mass	Main ions
2-Butanone	C_4_H_8_O	72.0575	43.2, 57.1, 72.1
Isobutanol	C_4_H_10_O	74.0732	39.3, 41.2, 42.3, 43.2, 55, 56.2, 57.1, 74.2
4-Methyl-2-pentanone	C_6_H_12_O	100.0888	39.3, 41.3, 43.1, 57.3, 58.2, 85.1, 100.1
2-Hexanone	C_6_H_12_O	100.0888	39.2, 41.3, 43.1, 57.3, 58.1, 71.1, 85.2, 100.1

From the standard chemical mixture, a 20 μg/mL solution was made up in methanol (HPLC grade). We spiked 1 μL of the solution into a sorbent tube, which was then flushed for 3 min with nitrogen at a flow rate of 100 mL/min using a Solution Loading Rig (Markes International Limited, UK). These tubes are then analysed by the instruments.

We measure the stability of the instruments over a four week ‘*cycle*’. At the beginning of each cycle, a batch of spiked tubes are prepared, these tubes were stored at 4 °C and analysed by the instruments over the four weeks – we will call these tubes ‘*stored*’ samples. Another tube is also prepared by using a freshly opened vial of the standard chemical mixture on each day of analysis and analysed on the day – we will call these tubes ‘*fresh*’ samples. Table S2 shows the scheduled analyses of the tubes over the four week cycle. On each day of analysis, two fresh samples followed by two stored samples were run in the morning and the same samples in the afternoon, these were the only samples ran on those days.

### 2.3 Thioethers stability study setup

The GC-QTOF instrument was used to measure the stability of the thioethers on sorbent tubes. We designed the experiment in the following manner:

A combined 0.3 ppm allyl methyl sulphide and 1-methylthio-propane, and 1.6 ppm (E)-1-methylthio-1-propene and (Z)-1-methylthio-1-propene solution was made up in methanol (HPLC grade). We spiked 1 μL of the solution into a sorbent tube, which was flushed in the same manner as Section 2.2. The tubes were then stored at the following temperatures: 6.3 °C, 30.0 °C, 40.3 °C, 51.3 °C and 60.5 °C in incubators or ovens.

The compound allyl methyl sulphide was purchased from Sigma-Aldrich (Belgium) and 1-methylthio-propane from ABCR GmbH & Co. (Karlsruhe, Germany). (E)-1-methylthio-1-propene and (Z)-1-methylthio-1-propene were synthesized by Advanced Molecular Technologies Pty Ltd.(Melbourne, Australia).

After some initial experimentation, we scheduled the sampling time for each storage temperature (Table S3) to enable us to obtain a linear decrease in the biomarker concentration.

For each compound at a given temperature, the reaction rates, *k*, were calculated using the following the Differential Rate Law:

(1) $$\ln\frac{N}{N_{0}}=-kt,$$

where *N* is the peak area of a compound at a given time, *N*_0_ is the peak area of the compound at time 0, *k* is the reaction time, and *t* is the time in days.

The reaction rate, *k*, has a temperature dependency, which is usually given by the Arrhenius equation

(2) $$k=A\exp\left(-\frac{E_{a}}{RT}\right),$$

where *A* is the Arrhenius factor (days^−1^), *E*_*a*_ is the energy activation (kJ/mol), *T* is the absolute temperature in Kelvin, and *R* is the universal gas constant (8.314 J ⋅K^−1^ ⋅mol^−1^).

To find *k* at any given temperature, we first calculate *k* for all the storage temperatures used in the experiment. Re-arranging Eq. (2) to

(3) $$\ln k=-\frac{E_{a}}{R}\left(\frac{1}{T}\right)+\ln A,$$

which is the equation of a straight line *y* = *ax* + *b*. By plotting $\frac{1}{T}$ vs. ln*k*, and fit a line through the data points, we can calculate the value of *k* at any given temperature, thus find the values of *E*_*a*_ and *A*.

### 2.4 Data analysis

The data from both the instrument stability and the chemical stability studies were analysed to find the total amount of the compound presented in each sample. For the samples measured by the GC-MS instrument, we used MS Data Review, Version 8.2 and for the GC-QTOF data we used Mass Hunter Qualitative analysis, Version B.07.00. We took the following procedure with both software:

1. Extract the chromatogram of the known ion masses.
2. Look for peaks eluting in the retention time order given by the Supelco Certificate of Analysis for the calibration mix.
3. Validate that the peaks found are from the correct compound using the NIST library.
4. Use the ion extraction technique from the software to calculate the peak area.

## 3 Rationale for instrument stability setup

Preparing fresh samples for each day of analysis is both time consuming and cost ineffective for most laboratories, thus, it will be more convenient and much cheaper to use stored samples of the same chemicals to compare measurements^1^ across different days. To be able to use stored samples, we need to know the decay characteristics of these chemicals when stored over a period of four weeks. By comparing the relative amount measured by the instruments in both the fresh sample and stored sample analysed consecutively on each day will give us the decay of the stored samples, and understand how to use the stored tubes.

The experiment was designed (1) to see the day to day changes in the instruments with no other variability, and (2) to see the variability within a single day. We ran repetitions of the same samples on each day of analysis to capture the change in the measured values over a day. This will inform future decisions on whether samples analysed on the same day can be normalised by just one overall value, or by some interpolated values throughout the day. We assume in this instance that samples do not degrade in the 12 h between the first and last measurement of the day.

The tubes were only analysed on seven out of a possible 28 days for the entire four week cycle. This allowed the instruments to be used for other purposes, and also gave us the opportunity to see the changes in the instrument when different samples were analysed in between the standard chemicals, thus capturing other possible characteristics of the instrument over the cycle.

## 4 Results and discussion

### 4.1 GC-MS results

**Fig. 1:**
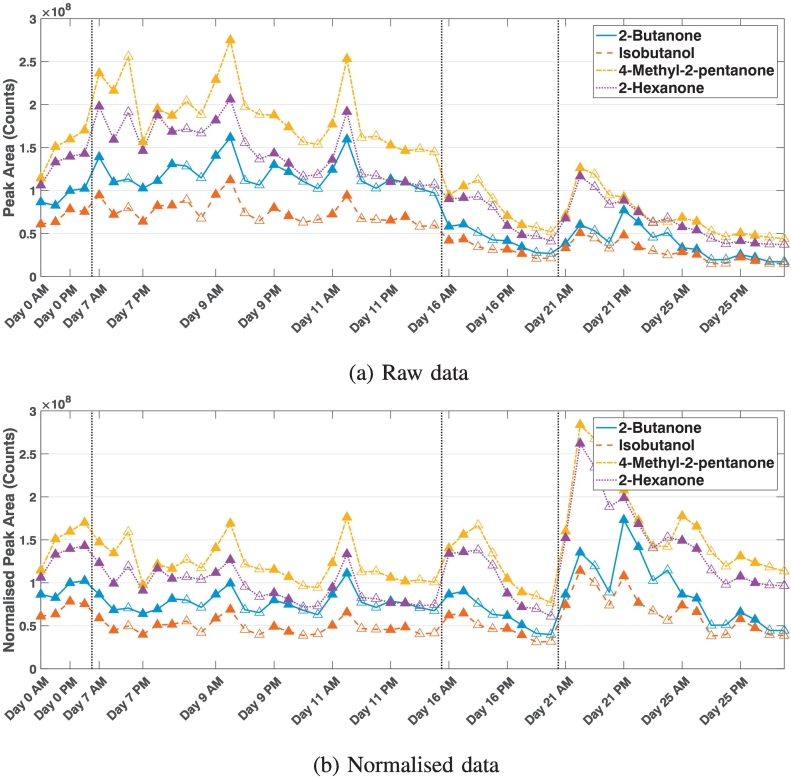
The four standard chemicals (a) measured by the GC-MS instrument over a period of four weeks, and (b) as normalised by the first measurement of 2-hexanone of each day of analysis in the GC-MS. The closed data points show fresh samples, and the open data points are the stored samples from Day 0. The vertical lines separate the weeks.

**Fig. 2:**
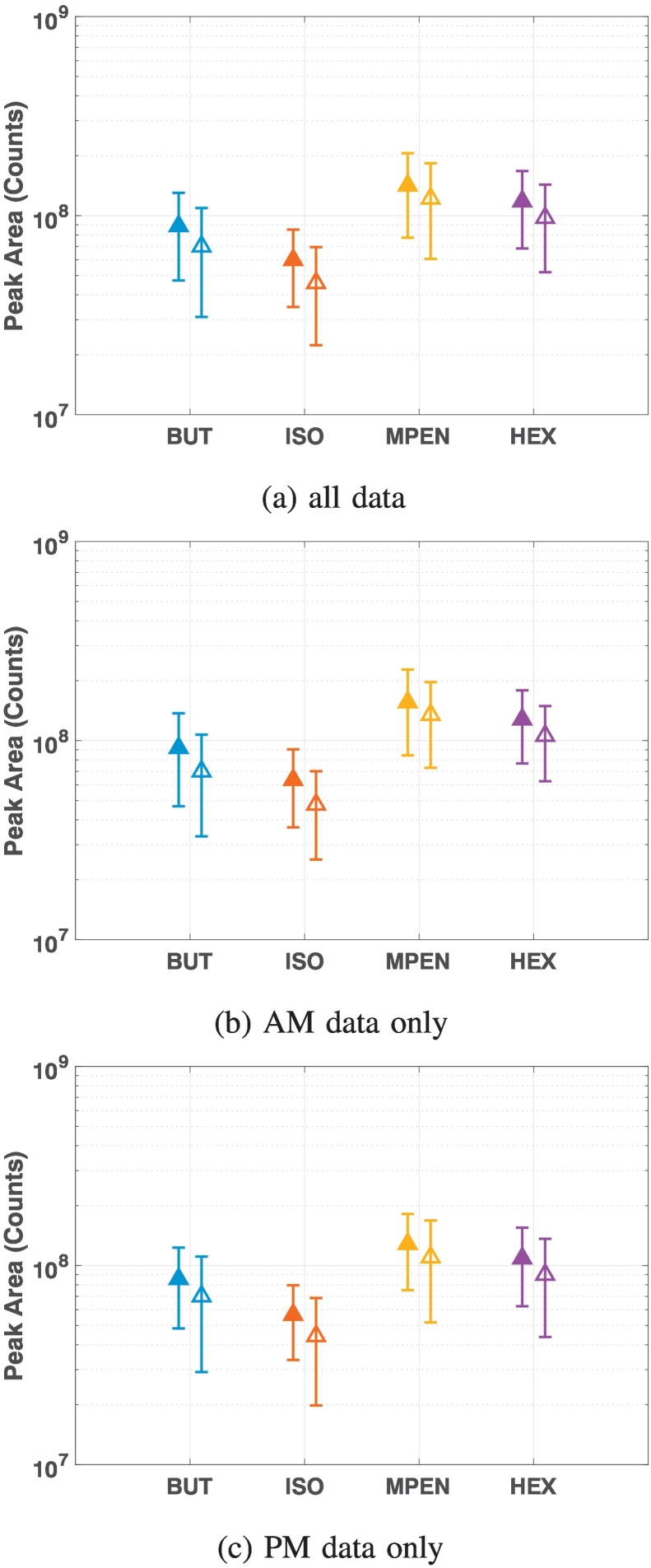
The mean and standard deviation of the four standard chemicals measured over the four week cycle for the GC-MS instrument. The closed data points show fresh samples and the open data points are the stored samples from Day 0.

For the GC-MS instrument, only one cycle of data was obtained due to lab requirement. We observe the following features in the data (Fig. 1a): (1) The four chemicals follow a similar pattern over the course of four weeks. (2) There is a large variation in chemical levels as measured by the instrument from day to day, demonstrating there is a definite ‘day of analysis' effect with the instrument. (3) The day of analysis effect is irrespective of whether the tube was stored from Day 0 or spiked freshly on the day, both the fresh and stored samples have similar standard deviations in their measured values over the four weeks (Fig. 2). (4) There is also considerable variation of measured values during the day, this can not be attributed to the degradation of the samples during the day as from Fig. 1a we can see the values do not always decrease over a day (e.g. the values increased on Day 0), and the standard deviation of the samples are not always larger in the afternoon. (5) There is in general a larger variation in measurements from the afternoon than measurements from the morning (Fig. 2), indicating the instrument's response changes as samples are run over a day.

The large day of analysis effect in the GC-MS data means that some normalisation on the data will need to be performed when comparing samples that are measured over a long period of time. The data analysed on the same day can be normalised by one overall factor, or by analysing more than one standard in the day of analysis and thus interpolate normalisation factors for every measurement during the day. We do not believe the second option is valid in this case because the measured values for a chemical do not change linearly over a day (Fig. 1a), and due to the length of the run time it is not practical to have more than two standards run in a day.

We choose to use the first measurement of the day as the normalisation factor for each day because there is no overall trend of the measurements in one day. The data is thus normalised as follows:

(4) $$X_{ij}^{\prime }=\frac{X_{ij}}{C_{i}}\times C_{1},$$

where *i* is the day of analysis, *j* is the sample number, $X_{ij}^{\prime }$ and *X*_*ij*_ are the normalised and raw measurements respectively, *C*_*i*_ is the normalisation factor on day *i* and *C*_1_ is the normalisation factor on Day 1 of analysis. The value *C*_1_ is included in the normalisation so all values are on the same magnitude as raw measurement.

The standard deviation of the data after normalisation (Table S4), allows us to compare the normalised data. We found the data normalised by 2-hexanone has the smallest standard deviation on average. Comparing the data normalised by 2-hexanone (Fig. 1b) to the raw data (Fig. 1a) we can see the normalised data is a lot more consistent over the four week period, although there remains a large variation for the Monday of week 4 (Day 21). The unusual behaviour of the instrument on Day 21 could be due to a power failure on Friday the previous week (i.e. Day 18)^2^. The large variation within the normalised data is also evident in Table S4. This means cautions need to be observed when presenting GC-MS data; for example, if data presented showing two groups of samples has a smaller difference than the variation observed when measuring the same sample during a day (in this case, about 10% of the measured value), then the result can’t be presented as significant.

### 4.2 GC-QTOF-MS results

For the GC-QTOF-MS instrument, we measured the standards over two cycles in sequence, i.e. data from eight consecutive weeks. While we aimed to keep the analysis days consistent with the schedule from Table S2, circumstances inevitably change, causing some variations in the schedule.

**Fig. 3:**
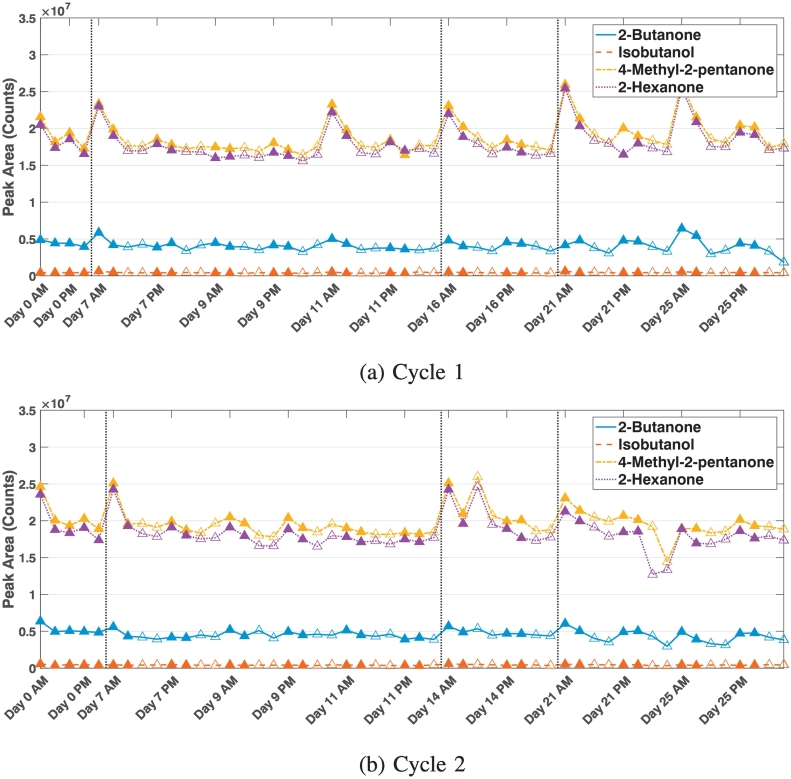
The four standard chemicals measured by the GC-QTOF-MS instrument for the first (top) and second (bottom) cycle over a period of four weeks. The closed data points show fresh samples, and the open data points are the stored samples from Day 0. The vertical lines separate the weeks.

**Fig. 4:**
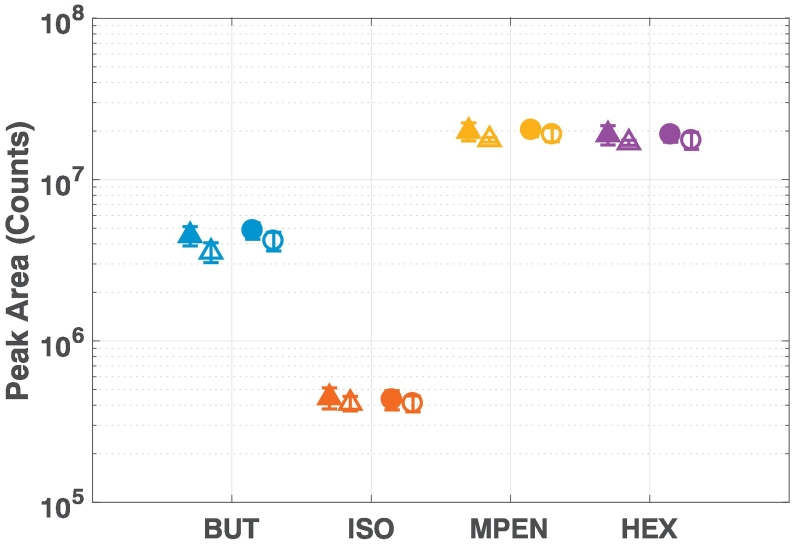
The mean and standard deviation of the four standard chemicals measured during Cycles 1 (triangle) and 2 (circle) for the QTOF instrument. The closed data points show fresh samples and the open data points are the stored samples from Day 0. See Fig. S1 for statistics of the data from AM or PM only.

Comparing the results here with the GC-MS instrument we notice several differences in the data: firstly, the peak areas of each chemical as measured by the GC-QTOF-MS do not change dramatically over the four weeks (Figs. 3 and 4). We observe the fold change between maximum and minimum peak areas over the eight weeks is approximately three times smaller than those from the GC-MS instrument (Table 2). This shows the QTOF instrument is a lot more stable than the GC-MS instrument, and raw measurement taken over a short period of time could be compared with each other.

**Table 2:** Fold change between maximum and minimum peak areas measured by the mass analysers over the cycles.

		BUT	ISO	MPEN	HEX
GC-MS cycle 1	All	9.40	7.63	6.27	5.52
	Fresh	7.32	6.12	5.77	5.34
	Stored	7.47	6.06	5.83	5.13
GC-QTOF both cycles	All	4.26	2.08	2.03	2.22
	Fresh	1.91	1.81	1.75	1.83
	Stored	3.86	1.87	1.86	2.00

The second feature we notice from Fig. 3 is that the four chemicals have very different values. The values for 4-methyl-2-pentanone and 2-hexanone are almost an order of magnitude higher than those of 2-butanone, which is again an order of magnitude higher than those from isobutanol. This difference in values between the chemicals is not observed in the GC-MS analysis, where the values are in the same order of magnitude, even though the samples were prepared in exactly the same procedure. The differences in EPA could be associated to the way the-time-of-flight operates and the use of constant pressure in the QTOF across the chromatographic run. Constant pressure gives different column flows at different oven temperatures. A different flow can give a different response for the same amount of a particular analyte or a different limit of detection. Therefore, it is important quantitative standards are run on each machine if quantitative values are reported.

Thirdly, we observed that the first measurement of the day (a fresh sample) almost always had a much higher value than the rest of the samples measured on the same day – on average the first samples can be between 4% to 14% higher in measured peak areas than the samples from the rest of the day for the four chemicals. As we measure a second fresh sample immediately after the first one and it is much more similar to the rest of the day's samples, we can conclude this phenomenon is not due to a fresh sample being measured but rather a stability issue. This indicates that the first sample of any day of analysis is not reliable and shouldn’t be used for any analysis. It is therefore advisable to measure a blank or empty tube sample as the first measurement of the day in the QTOF.

Even though the data here show measurement taken over short period of time with the GC-QTOF instrument could be compared with each other, we still recommend a tube with standard chemicals is analysed at the start of each day's analysis so measurements over long periods can be compared. We again recommend normalising each day's measurements using one normalisation factor because the measurements do not change linearly over a day. We normalised the data using either the first or the second measurement (due to the abnormal value of the first measurement) of each of the four standard chemicals of each day as the normalisation factor using Eq. (4) (Table S5). We observed the data normalised using the second measurement has similar variability as the raw samples. Further, we note that as with the GC-MS data, the normalisations using 4-methyl-2-pentanone and 2-hexanone achieve smaller standard deviations in the data. The variability in the normalised data is approximately 10% of the measured values, showing caution also needs to be observed when presenting results from GC-QTOF instruments.

We recommend the following procedure for analysing GC-QTOF data: (1) Measure a blank or empty tube sample as the first measurement of the day; (2) measure a tube of standard chemicals to be used for normalisation; (3) measure the rest of the samples. The measurement taken over short period of time with the GC-QTOF instrument could be compared with each other. When comparing data over a long period of time, we recommend to normalise each day's data using the 4-methyl-2-pentanone or 2-hexanone as the normalisation factor.

### 4.3 Comparison between mass analysers

We designed our stability experiment for the two mass analysers using the same schedule and standard chemicals in order to compare the results between the instruments. We observed the following when comparing the results: (1) the measured values of the standard chemicals are different between the two instruments, which means we can not compare measurements from different mass analysers; (2) the heavier compounds 4-methyl-2-pentanone (MW = 100) and 2-hexanone (MW = 100) give better normalised results for both instruments; (3) the normalised results has a variation approximately 10% of the measured values for both instruments, thus need to be taken into account when presenting significant findings from these instruments.

A solvent delay time of 2 min was used to enable the capture of the fast eluting compounds, 2-butanone and isobutanol. Ideally a solvent delay of 4 min is need to reduce damage to the source filaments from the solvent (methanol). Therefore, we recommend to measure a standard tube containing only 4-methyl-2-pentanone and 2-hexanone.

It is also possible to spike every sample for analysis with some standard chemicals (commonly referred to as ‘internal standards'). As these chemicals are mixed with methanol before spiking, a solvent delay time of at least 3 min will need to be introduced. Such delay time means the low molecular weight compounds from the breath sample could be lost, thus we did not study the use of internal standards for breath samples. Alternatively, the internal standards can be injected in gas form to avoid solvent delay or remove ions characteristic of methanol prior to measurement, this will be subject to future studies.

### 4.4 Chemical stability on sorbent tubes

#### 4.4.1 EPA standards

We used both fresh and stored EPA standard chemicals to understand the day of analysis effects of each mass analyser instrument. By comparing the relative amount measured by the instruments in both the fresh and stored samples, we can determine whether the stored samples have decayed over the four week period and thus understand how to use the stored samples.

For both instruments, we observe the fresh samples always had slightly higher measured values than the stored samples (Figs. 2 and 4). This effect was observed in the samples analysed in the morning and those analysed in the afternoon. This difference in measured values between the fresh and the stored samples is smaller than the overall change in measured peak areas over the four week cycle (Fig. 1a). Moreover, the fold changes between maximum and minimum peak areas are not significantly different between fresh and stored samples (Table 2). Thus the difference between fresh and stored samples could be due to the instrument's response over the day as the stored samples were always analysed after the fresh samples. We therefore can not conclude that there is clear evidence to suggest that the stored samples of these chemicals degrade over the course of the cycle. This means we can prepare these standard samples at the beginning of a four week period and measure the stored samples on the day of analysis, and use the resulting values of the chemicals to standardise the other samples so they can be compared across different days of analysis.

#### 4.4.2 Breath specimens: a real world case scenario

The EPA standard chemicals used in our stability study showed no obvious signs of degradation over the period of four weeks, however, this is not the case for some breath volatiles. Thioethers, previously found in the breath of adults as indicative of early malaria infection [17], are very unstable therefore, it is important to understand their decay characteristics in sorbent tubes. This will allow us to (1) recommend optimal storage times and temperatures for the breath sample between collection and analysis; and (2) estimate the concentration of the thioethers at the time of collection from the storage time.

**Fig. 5:**
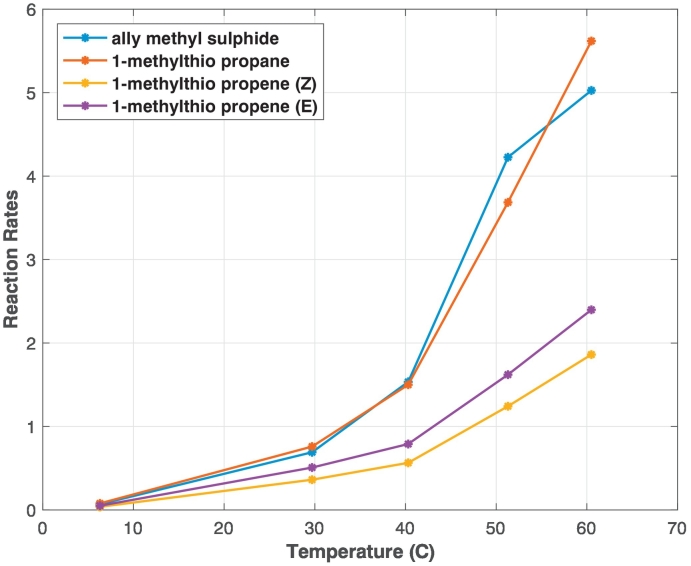
Reaction rate, *k*, for various storage temperatures of the four thioethers. See data in [19] for exact values.

From our experiment, we were able to determine the rate at which the thioethers decreased at several temperatures. For example, we found that the 1-methylthio-propane level falls to 10% of the original concentration within 3 days for temperatures near 30 °C and 34 days with a temperature of 6.3 °C. We calculated the reaction rates, *k*, for each storage temperature according to Eq. (1) (Fig. 5). The reaction rates show that 1-methylthio-propane was the compound with the highest decay rates, meaning that compared to other thioethers it will degrade quicker on the sorbent tubes.

**Fig. 6:**
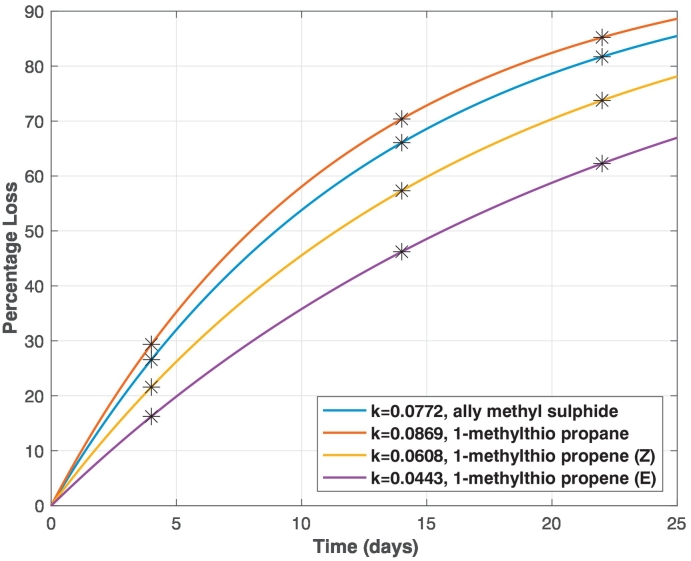
Percentage loss for each thioether in sorbent tubes stored at 6.5 °C for up to 25 days. The loss at key storage days of 4, 14 and 22 days are marked on the plots.

The reaction rates were then used to find the respective Arrhenius equations (Eq. (3)) for each compound (Table S6). The equations allow us to calculate *k* for any other temperature. For example, if we aim to store samples at 6.5 °C, we can calculate *k* for each of the thioethers, then estimate the loss of each compound after a given number of days using Eq. (1) (Fig. 6). From these estimates, 1-methylthio-propane will lose more than 85% of its original content after 22 days, i.e. only 15% of the compound would be still present on the sorbent tubes.

Our results of thioether decay characteristic suggest that there is a need to set a target time from breath collection to analysis by GC-MS. Further, the samples need to be kept at a low temperature to ensure the appropriate amount of the thioethers are still available in the sorbent tubes for analysis. In order to detect the thioethers, we therefore recommend that breath samples should be stored for no more than 14 days at 6.5 °C before analysis. At 14 days of storage the loss will be between 46% and 66% (Fig. 6), still within detectable levels of the mass analyser.

The reaction rates also enable the estimation of the original concentration at the time of collection using Eq. (1) (Fig. S2). By estimating the original concentration, it allows us to make reliable comparisons between the samples collected and analysed at different times.

In previous studies, Mochalski et al. [18] showed volatile sulphur compounds decay very quickly in sorbent tubes, however, their study did not specifically analyse the four thioethers we studied here. Harshman et al. [16] studied the effect of storage in sorbent tubes on 74 VOCs in exhaled breath. In their study, three of the four thioethers: allyl methyl sulphide, 1-methylthio-propane and (E)-1-methylthio-1-propene, were monitored and their results indicate there was no decay in thioether levels when sorbent tubes were stored at 4 °C, small changes at 21 °C and moderate changes at 37 °C (Table S7). Moreover, Harshman et al.’s results suggest there is a positive enrichment of thioethers when stored at ambient temperature for short time, although the authors only discussed the negative enrichment effect for sulphur compounds. We used Arrhenius equations to estimate the relative concentrations for the three thioethers at 4, 21 and 37 °C, and for 3, 14 and 31 days of storage.

Our data shows higher loss at the same temperatures and days of storage, and no positive enrichment of the compounds (Table S7), probably due to the following experimental differences: (1) we used a mixture of pure compounds (thioethers) dissolved in methanol, while [16] measured the decay of VOCs in exhaled breath. (2) There is a difference in concentration of sulphur compounds in the sample. In our study, we spiked the sorbent tubes with thioethers at the ppm level (see Section 2.3) in methanol and that could have created chemical reactions on the sorbent tubes over time. These levels are above what would be normally found in healthy individuals [20]. (3) In [16] the sampling bags that were used to collect the exhaled breath before being concentrated onto sorbent tubes, whereas in our experiment the compounds were spiked directly onto the sorbent tubes. The authors of [16] have noted that sampling bag related artifacts can contribute to the observed results.

These results show that stability of breath volatiles on sorbent tubes is affected by a number of factors (bags, sorbent tube materials, cold trap, VOCs mixture, concentration, etc.) and they have utility only when tested under the conditions at which they will be used. Furthermore, the environment also plays a role in stability as can be seen from the difference between lab and field results in [16].

## 5 Conclusion and future work

We show in this paper detailed study of two critical factors in breath analysis research using gas chromatography-mass spectrometry instruments: the stability of the instrument and the stability of specific VOCs on sorbent tubes.

We used a set of commercially available standard chemicals to study the instrument stability. We found the single-quadrupole GC-MS instrument has large variation in the measured values of the same sample within a single day and within the four week period in which we performed the experiment, while GC-QTOF-MS instrument is a lot more stable over the same period. Further, we found the first measurement of the day from the GC-QTOF-MS instrument is often very different from the rest of the measurements. Our findings suggest that before comparing data from any gas chromatography instrument, the measurements first need to be normalised. We recommend a run schedule of the instrument to include an empty tube, followed by a standard chemicals tube at the beginning of each day. We recommend the use of the following chemical standards 2-hexanone and 4-methyl-2-pentanone to normalise each day's measurements.

For both instruments we observed standard deviation of the normalised measurement is approximately 10% of the measured values. This indicates caution needs to be observed when presenting significant findings from these instruments.

When comparing the measurements between the two mass analysers, we found not only the measured values are very different, there is also a difference in the ratio of the measured compound. Therefore, our findings between the two mass analysers suggest there is no clear way to compare data from different instruments. We also suggest small studies of instrument stability be performed before studies of breath sample to ensure appropriate normalisation.

In future work, we aim to repeat the instrument stability experiment to study the long term trend in the behaviour of the GC instruments. We will also study the use of internal standards (breath samples spiked with standard chemicals) for the instruments.

We studied the stability of the standard chemicals on sorbent tubes and found there is no clear evidence to suggest that these chemicals degrade over the four week period, thus these standards can be prepared at the beginning of a four week period for use on each day of analysis.

We further studied the chemical stability of four sulphur compounds, thioethers, that were previously shown to be indicative of malaria infection. Our findings suggest that these compounds need to be stored at a low temperature, 6 °C, for no more than 20 days to avoid the total decay of the chemicals. Our results also allows us to calculate the approximate level of the chemicals on the day of collection.

In future work, we aim to study the stability of thioethers in a complex matrix (i.e. under exhaled breath background), in both lab and field conditions and compare with our current results. The comparison of stability in different backgrounds will allow a deeper understanding of how other volatiles affect the decay rate and the implications on the stability of thioethers. We will also collect multiple samples from the same volunteers over a period of time. This type of study will inform us of reproducibility and repeatability of results for real breath samples. Such results would strengthen the discovery of biomarkers in breath samples for diagnosis.

## Research data

The processed and raw data for this paper are available online [19,21].

## Funding

This work was supported by the Bill & Melinda Gates Foundation Grant OPP1142041 and the Commonwealth Scientific and Industrial Research Organisation (CSIRO). The funding organisations had no involvement in the study design, data collection and analysis, writing the report, or in the decision to submit the article for publication.

## Footnotes

1 In this paper, we use the term ‘measurement’ to indicate the analysis of a single sample.

2 There was a second power failure on Day 22, however with other samples run on Days 23 and 24 we do not see a similar unusual behaviour on Day 25.
