# Supplementary material for: Stability control for breath analysis using GC-MS
Source: J Chromatogr B Analyt Technol Biomed Life Sci. 2018 Oct 15;1097-1098:27–34. doi: 10.1016/j.jchromb.2018.08.024 (PMC6167955; doi:10.1016/j.jchromb.2018.08.024)
Supplement: Supplementary file 1 — Supplementary material [file mmc1.pdf]

Stability control for breath analysis using GC-MS: Supplementary Materials

X. Rosalind Wang[a,*], Julie Cassells[b], Amalia Z. Berna[b,c]

[a] *CSIRO Data61, PO Box 76, Epping, NSW 1710, Australia*
[b] *CSIRO Health and Biosecurity, GPO Box 1700 Canberra, ACT 2601, Australia*
[c] *Department of Pediatrics, Washington University School of Medicine, St. Louis, MO 63110 USA.*

Table S1: Approximate retention time of the standard chemicals used for instrument stability.

Compound	Approx. RT (min)	
	GC-MS	GC-QTOF
2-Butanone	2.21	2.59
Isobutanol	3.92	2.86
4-Methyl-2-pentanone	4.3	4.65
2-Hexanone	5.92	6.26

Table S2: Schedule for one cycle of the experiment. The 'stored' samples are all prepared together on Monday of week 1, stored and analysed across the four weeks as shown. The 'fresh' samples are prepared on the day of the analysis.

	Week 1	Week 2			Week 3	Week 4	
	Mon	Mon	Wed	Fri	Mon	Mon	Fri
Fresh	x	x	x	x	x	x	x
Stored		x	x	x	x	x	x

*Corresponding author
Email addresses: Rosalind.Wang@csiro.au (X. Rosalind Wang), a.berna@wustl.edu (Amalia Z. Berna)

Preprint submitted to Journal of Chromatography B *August 24, 2018*

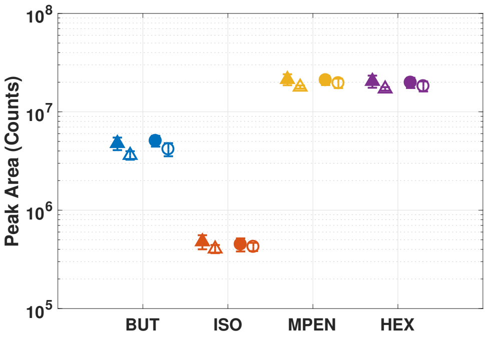

(a) AM data only

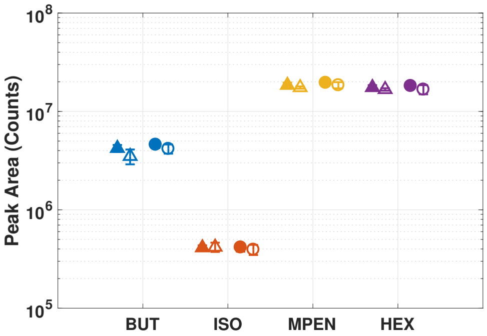

(b) PM data only

Figure S1: The mean and standard deviation of the four standard chemicals measured during Cycle 1 (triangle) and 2 (circle) for the QTOF instrument for AM or PM data only. The closed data points show fresh samples and the open data points are the stored samples from day 0.

Table S3: Sampling times for thioethers during stability trial.

	Storage time (days)							
	0	1	2	3	6	27	28	34
$6.3°C$	2	2	2	2	1	1	1	1
$29.7°C$	3	3	3	1				
$40.3°C$	3	3						
$51.3°C$	3							
$60.5°C$	3							

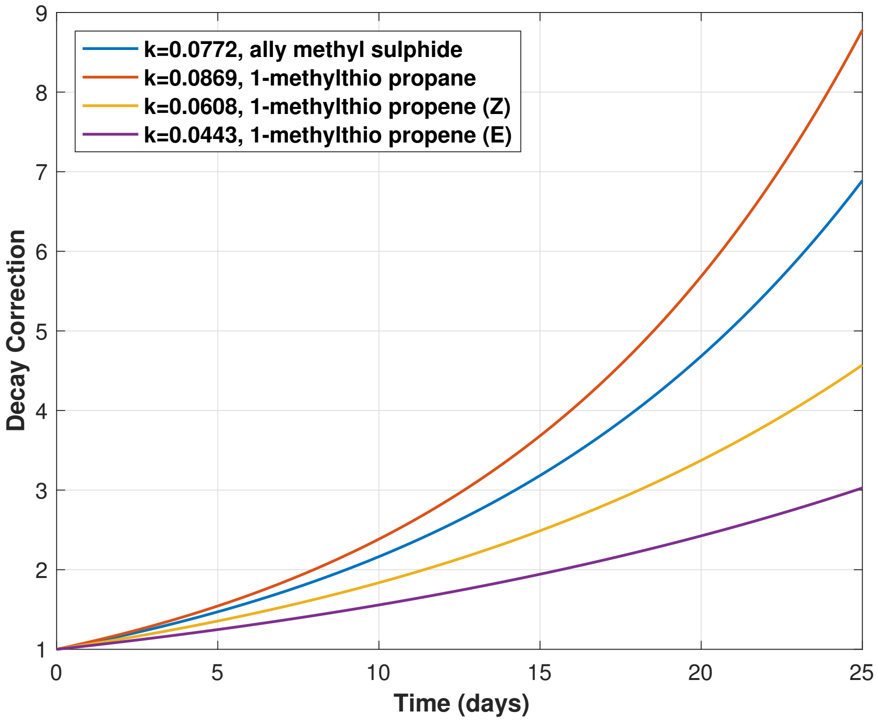

Figure S2: Decay correction for each thioether in sorbent tubes stored at $6.5°C$ for up to 25 days. The graph shows the multiplication factor at each day of analysis to obtain the count of each thioether at time of collection.

Table S4: The standard deviation of normalised and raw measurements of the four standard chemical: 2-butanone (BUT), isobutanol (ISO), 4-methyl-2-pentanone (MPEN) and 2-hexanone (HEX). Each column represent data normalised by the measured values of a chemical from the first measurement of the day.

Analysed Compounds	Sample Type	Raw values	Compound used for Normalisation			
			BUT	ISO	MPEN	HEX
BUT	fresh	4.14×10^7	2.55×10^7	2.23×10^7	2.12×10^7	2.17×10^7
	stored	3.91×10^7	2.08×10^7	1.94×10^7	1.63×10^7	1.79×10^7
ISO	fresh	2.52×10^7	1.76×10^7	1.35×10^7	1.44×10^7	1.35×10^7
	stored	2.36×10^7	1.49×10^7	1.20×10^7	1.07×10^7	1.07×10^7
MPEN	fresh	6.42×10^7	3.92×10^7	3.17×10^7	3.10×10^7	3.10×10^7
	stored	6.13×10^7	4.10×10^7	3.17×10^7	2.84×10^7	2.70×10^7
HEX	fresh	4.96×10^7	3.95×10^7	2.96×10^7	2.98×10^7	2.75×10^7
	stored	4.56×10^7	4.05×10^7	2.94×10^7	2.73×10^7	2.41×10^7
averages		4.37×10^7	2.99×10^7	2.37×10^7	2.24×10^7	2.17×10^7

Table S5: The standard deviation of normalised and raw measurements of the four standard chemical for both cycles of QTOF experiment. We normalise the data using the first or the second sample of the day. Each column represent data normalised by a chemical measured on the day.

QTOF Cycle	Sample No. for norm.	Compound used for Normalisation				Raw values
		BUT	ISO	MPEN	HEX	
1	1	1.64×10^6	1.22×10^6	1.23×10^6	1.40×10^6	9.47×10^5
	2	1.15×10^6	1.14×10^6	9.68×10^5	9.76×10^5	
2	1	1.37×10^6	1.56×10^6	1.45×10^6	1.44×10^6	1.13×10^6
	2	1.37×10^6	1.38×10^6	1.08×10^6	1.08×10^6	

Table S6: Arrhenius equations for the four thioethers: allyl methyl sulphide (AMS), 1-methylthio-propane (MTP), (E)-1-methylthio-1-propene (MTPN-E) and (Z)-1-methylthio-1-propene (MTPN-Z).

Compound	Equation
AMS	$\ln k = -7659.1\frac{1}{T} + 24.829$
MTP	$\ln k = -7422.8\frac{1}{T} + 24.1$
MTPN-E	$\ln k = -6687.3\frac{1}{T} + 20.797$
MTPN-Z	$\ln k = -6626.7\frac{1}{T} + 20.897$

Table S7: Comparison of relative concentration, N/N_0, at 3, 14 and 31 days between our results and those reported in Harshman *et al.* [1].

		Our study			Harshman *et al.*		
Compound	Temp ($^{\circ}C$)	day 3	day 14	day 31	day 3	day 14	day 31
AMS	4	0.8345	0.4299	0.1542	-	-	-
	21	0.4092	0.0155	0.0001	1.0489	0.7358	0.5044
	37	0.0326	0.0000	0.0000	0.7817	0.4413	0.1857
MTP	4	0.8145	0.3839	0.1200	-	-	-
	21	0.3812	0.0111	0.0000	1.0638	0.9183	0.747
	37	0.0289	0.0000	0.0000	0.875	0.7616	0.5055
MTPNE	4	0.8984	0.6064	0.3304	-	-	-
	21	0.6490	0.1330	0.0115	1.1043	0.9164	0.6731
	37	0.2474	0.0015	0.0000	0.9262	0.7764	0.433

References

[1] S. W. Harshman, N. Mani, B. A. Geier, J. Kwak, P. Shepard, M. Fan, G. L. Sudberry, R. S. Mayes, D. K. Ott, J. A. Martin, C. C. Grigsby, Storage stability of exhaled breath on tenax ta, Journal of Breath Research 10 (4) (2016) 046008.
URL http://stacks.iop.org/1752-7163/10/i=4/a=046008
